# Two-Stream Attention Network for Pain Recognition from Video Sequences

**DOI:** 10.3390/s20030839

**Published:** 2020-02-04

**Authors:** Patrick Thiam, Hans A. Kestler, Friedhelm Schwenker

**Affiliations:** 1 Institute of Medical Systems Biology, Ulm University, Albert-Einstein-Allee 11, 89081 Ulm, Germany; patrick.thiam@uni-ulm.de (P.T.); hans.kestler@uni-ulm.de (H.A.K.); 2 Institute of Neural Information Processing, Ulm University, James-Frank-Ring, 89081 Ulm, Germany

**Keywords:** convolutional neural networks, long short-term memory recurrent neural networks, information fusion, pain recognition

## Abstract

Several approaches have been proposed for the analysis of pain-related facial expressions. These approaches range from common classification architectures based on a set of carefully designed handcrafted features, to deep neural networks characterised by an autonomous extraction of relevant facial descriptors and simultaneous optimisation of a classification architecture. In the current work, an end-to-end approach based on attention networks for the analysis and recognition of pain-related facial expressions is proposed. The method combines both spatial and temporal aspects of facial expressions through a weighted aggregation of attention-based neural networks’ outputs, based on sequences of Motion History Images (MHIs) and Optical Flow Images (OFIs). Each input stream is fed into a specific attention network consisting of a Convolutional Neural Network (CNN) coupled to a Bidirectional Long Short-Term Memory (BiLSTM) Recurrent Neural Network (RNN). An attention mechanism generates a single weighted representation of each input stream (MHI sequence and OFI sequence), which is subsequently used to perform specific classification tasks. Simultaneously, a weighted aggregation of the classification scores specific to each input stream is performed to generate a final classification output. The assessment conducted on both the *BioVid Heat Pain Database (Part A)* and *SenseEmotion Database* points at the relevance of the proposed approach, as its classification performance is on par with state-of-the-art classification approaches proposed in the literature.

## 1. Introduction

An individual’s affective disposition is often expressed throughout facial expressions. Human beings are therefore able to assess someone’s current mood or state of mind by observing his or her facial demeanour. Therefore, an analysis of facial expressions can provide some valuable insight about one’s emotional and psychological state. Thus, facial expression recognition (FER) has been attracting a lot of interest from the research community in the recent decades and constitutes a steadily growing area of research, particularly in the domains of machine learning and computer vision. The current work focuses on the analysis of facial expressions for the assessment and recognition of pain in video sequences. More specifically, a two-stream attention network is designed, with the objective of combining both temporal and spatial aspects of facial expressions, based on sequences of motion history images [1] and optical flow images [2], to accurately discriminate between neutral, low, and high levels of nociceptive pain. The current work is organised as follows. An overview of pain recognition approaches based on facial expressions is provided in Section 2, followed by a thorough description of the proposed approach in Section 3. In Section 4, a description of the datasets used for the assessment of the proposed approach as well as the performed experiments is provided, followed by a description of the corresponding results. The current work is subsequently concluded in Section 5 with a short discussion and description of potential future works.

## 2. Related Work

Recent advances in both domains of computer vision and machine learning, combined with the release of several datasets designed specifically for pain-related research (e.g., *UNBC-McMaster Shouder Pain Expression Archive Database* [3], *BioVid Heat Pain Database* [4], *Multimodal EmoPain Database* [5] and *SenseEmotion Database* [6]), have fostered the development of a multitude of automatic pain assessment and classification approaches. These methods range from unimodal approaches, characterised by the optimisation of an inference model based on one unique and specific input signal (e.g., video sequences [7,8], audio signals [9,10] and bio-physiological signals [11,12,13]), to multimodal approaches that are characterised by the optimisation of an information fusion architecture based on parameters stemming from a set of distinctive input signals [14,15,16].

Regarding pain assessment based on facial expressions, several approaches have been proposed, ranging from conventional supervised learning techniques based on specific sets of handcrafted features, to deep learning techniques. These approaches rely on an effective preprocessing of the input signal (which in this case consists of a set of images or video sequences) and involves the localisation, alignment and normalisation of the facial area in each input frame. Moreover, further preprocessing techniques include the localisation and extraction of several fiducial points characterising specific facial landmarks, and in some cases, the continuous extraction of facial Action Units (AUs) [17,18]. The preprocessed input signal, as well as the extracted parameters, are subsequently used to optimise a specific inference model based on different methods. In [19], the authors use an ensemble of linear Support Vector Machines (SVMs) [20] (each trained on a specific AU), in which inference scores are subsequently combined using Logistical Linear Regression (LLR) [21] for the detection of pain at a frame-by-frame level. The authors in [22] apply a *k*-Nearest Neighbours (*k*-NN) [23] model on geometric features extracted from a specific set of facial landmarks for the recognition of AUs. Subsequently, the pain intensity in a particular frame is evaluated based on the detected AUs by using a pain evaluation scale provided by Prkachin and Solomon [24]. Most recently, the authors in [25] improve the performance of a pain detection system based on automatically detected AUs by applying a transfer learning approach based on neural networks to map automated AU codings to a subspace of manual AU codings. The encoded AUs are subsequently used to perform pain classification, using an Artificial Neural Network (ANN) [26].

In addition to AU-based pain assessment approaches, several techniques based on either facial texture, shape, appearance and geometry or on a combination of several of such facial descriptors have been proposed. Yang et al. [27] assess several appearance-based facial descriptors by comparing the pain classification performance of each feature with its spatio-temporal counterpart using SVMs. The assessed spatial descriptors consist of Local Binary Patterns (LBP) [28], Local Phase Quantization (LPQ) [29], Binarized Statistical Image Features (BSIF) [30] as well as each descriptor’s spatio-temporal counterpart extracted from video sequences on three orthogonal planes (LBP-TOP, LPQ-TOP and BSIF-TOP). In [8,31], the authors propose several sets of spatio-temporal facial action descriptors based on both appearance- and geometry-based features extracted from both the facial area, as well as the head pose. Those descriptors are further used to perform the classification of several levels of pain intensities using a Random Forest (RF) [32] model. Similarly, the authors in [7,14,15,33], propose several spatio-temporal descriptors extracted either from the localised facial area or from the estimated head pose, including, among others, Pyramid Histograms of Oriented Gradients (PHOG) [34] and Local Gabor Binary Patterns from Three Orthogonal Planes (LGBP-TOP) [35], to perform the classification of several levels of nociceptive pain. The classification experiments are also performed with RF models and ANNs.

Alongside handcrafted feature-based approaches, several techniques based on deep neural networks have also been proposed for the assessment of pain induced facial expressions. Such approaches are characterised by the joint extraction of relevant descriptors (from the preprocessed raw input data) and optimisation of an inference model, based on neural networks in an end-to-end manner. In [36,37,38], the authors propose a hybrid deep neural network pain detection architecture characterised by the combination of a feature embedding network consisting of a Convolutional Neural Network (CNN) [39] with a Long Short-Term Memory (LSTM) [40] Recurrent Neural Network (RNN), to take advantage of both spatial and temporal aspects of facial pain expressions in video sequences. Soar et al. [41] propose a similar approach by combining a CNN with a Bidirectional LSTM (BiLSTM), and using a Variable-State Latent Conditional Random Field (VRS-CRF) [42] instead of a conventional Multi-Layer Perceptron (MLP) to perform the classification. In [43], the authors also use a similar hybrid approach as in [36,37]; however, in this case, the feature embedding CNN is coupled to two distinct LSTM networks. The outputs of the LSTM networks are further concatenated and a MLP is used to perform the classification of the pain intensities in video sequences. Furthermore, Zhou et al. [44] propose a Recurrent Convolutional Neural Network (RCNN) [45] architecture for the continuous estimation of pain intensity in video sequences at the frame-level, whereas Wang et al. [46] propose a transfer learning approach, consisting of the regularisation of a face verification network, which is subsequently applied to a pain intensity regression task.

The current work focuses on the analysis of facial expressions for the discrimination of neutral, low and high levels of nociceptive pain in video sequences. Thereby, an end-to-end hybrid neural network characterised by the integration of spatial and temporal information at both the representational level of the input data (OFI and MHI) and the structural level of the proposed architecture (hybrid CNN-BiLSTM) is proposed. Furthermore, frame attention parameters [47] are integrated into the proposed architecture to generate an aggregated representation of the input data based on an estimation of the representativeness of each single input frame, in relation with the corresponding level of nociceptive pain. An extensive assessment of the proposed architecture is performed on both *BioVid Heat Pain Database (Part A)* [4] and *SenseEmotion Database* [6].

## 3. Proposed Approach

A video sequence can be characterised by both its spatial and temporal components. The spatial component represents the appearance (i.e., texture, shape and form) of each frame’s content, whereas the temporal component represents the perceived motion across consecutive frames due to dynamic changes of the content’s appearance through time. Most of the deep neural network approaches designed for the assessment of pain-related facial expressions generate spatio-temporal descriptors of the input data in two distinct and conjoint stages: a specific feature embedding neural network (which is commonly a pre-trained CNN) first extracts appearance based descriptors from the individual input frames (which are greyscale or colour images), and a recurrent neural network is subsequently used for the integration of the input’s temporal aspect based on sequences of previously extracted appearance features, thus generating spatio-temporal representations of video sequences that are used for classification or regression tasks. Therefore, both the temporal and spatial aspects of video sequences are integrated uniquely at the structural level (e.g., the architecture of the neural network) of such approaches. The current approach extends this specific technique by additionally integrating motion information at the representational level (e.g., input data) of the architecture throughout sequences of motion history images [1] and optical flow images [2].

### 3.1. Motion History Image (MHI)

Introduced by Bobick and Davis [48], a MHI consists of a scalar-valued image depicting both the location and direction of motion in a sequence of consecutive images, based on the changes of pixel intensities of each image through time. The intensity of a pixel in a MHI is a function of the temporal motion history at that specific point. A MHI $H_{\tau }$ is computed using an update function $\Psi \left(x,y,t\right)$, and is defined as follows,

(1) $$\begin{array}{c} H_{\tau }\left(x,y,t\right)=\left\{\begin{array}{cc} \tau & \mathrm{if}\;\Psi \left(x,y,t\right)=1\\ max\left(0,H_{\tau }\left(x,y,t-1\right)-\delta \right) & \mathrm{otherwise} \end{array}\right. \end{array}$$

where $\left(x,y\right)$ represents the pixel’s location, *t* the time and τ the temporal extent of the observed motion (e.g., the length of a sequence of images); $\Psi \left(x,y,t\right)=1$ is synonym of motion at the location $\left(x,y\right)$ and at the time *t*; and δ represents a decay parameter. The update function $\Psi \left(x,y,t\right)$ is defined as follows,

(2) $$\begin{array}{c} \Psi \left(x,y,t\right)=\left\{\begin{array}{cc} 1 & \mathrm{if}\;D\left(x,y,t\right)\geq \xi \\ 0 & \mathrm{otherwise} \end{array}\right. \end{array}$$

where ξ is a threshold; $D\left(x,y,t\right)$ represents the absolute value of the difference of pixel intensity values of consecutive frames and is defined as follows,

(3) $$D\left(x,y,t\right)=\left|I(x,y,t)-I(x,y,t\pm \Delta t)\right|$$

where $I\left(x,y,t\right)$ represents the pixel intensity at the location $\left(x,y\right)$ and at the time *t*; Δt represents the temporal distance between the frames.

**Figure 1:**
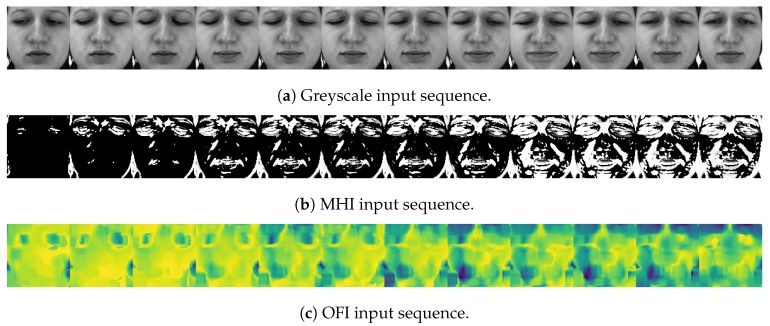
Data preprocessing. Following the detection, alignment, normalisation and extraction of the facial area in each frame of a video sequence, the images are converted into greyscale. MHI and OFI sequences are subsequently generated.

Therefore, the computation of a MHI consists in first performing image differencing [49] between a specific, preceding frame and the current *t*th frame, and detecting the pixel locations where a substantial amount of movement has occurred (depending on the value assigned to the threshold ξ) based on Equation (2). Subsequently, Equation (1) is used to assign pixel values to the MHI. If a motion has been detected at the location $\left(x,y\right)$ of the *t*th frame, a pixel value of τ is assigned at that location. Otherwise, the previous pixel value of that location is reduced by δ, thereby indicating the temporal occurrence of the motion at that specific location, according to the actual time *t*. This whole process is conducted iteratively until the entire sequence of images has been processed. The temporal history of motion is thereby encoded into the resulting MHI. Therefore, a whole sequence of images can be encoded into a single MHI. However, in the current work, a sequence of MHIs is generated from each single sequence of images by saving each single MHI generated at each single step of the iterative process described earlier. The resulting sequence of images is used as input for the designed deep neural networks. A depiction of such a sequence of MHIs can be seen in Figure 1b, with the corresponding sequence of greyscale images depicted in Figure 1a.

### 3.2. Optical Flow Image (OFI)

Optical flow refers to the apparent motion of visual features (e.g., corners, edges, textures and pixels) in a sequence of consecutive images. It is characterised by a set of vectors (optical flow vectors) defined either at each location $\left(x,y\right)$ of an entire image (dense optical flow [50,51]), or at specific locations of a predefined set of visual features (sparse optical flow [2,52]). The orientation of an optical flow vector depicts the direction of the apparent motion, whereas the magnitude of an optical flow vector depicts the velocity of the apparent motion of the corresponding visual feature in consecutive frames. Thus, an OFI provides a compact description of the location, direction and velocity of a specific motion occurring in consecutive frames. The estimation of the optical flow is based on the brightness constancy assumption, which stipulates that pixel intensities are constant between consecutive frames. If $I\left(x,y,t\right)$ is the pixel intensity at the location $\left(x,y\right)$ and at the time *t*, the brightness constancy assumption can be formulated as follows,

(4) $$I\left(x,y,t\right)=I\left(x+\delta x,y+\delta y,t+\delta t\right)$$

where $\left(\delta x,\delta y,\delta t\right)$ represents a small motion. By applying a first-order Taylor expansion, $I\left(x+\delta x,y+\delta y,t+\delta t\right)$ can be written as follows,

(5) $$I\left(x+\delta x,y+\delta y,t+\delta t\right)\approx I\left(x,y,t\right)+\frac{\partial I}{\partial x}\delta x+\frac{\partial I}{\partial y}\delta y+\frac{\partial I}{\partial t}\delta t.$$

Thus,

(6) $$\frac{\partial I}{\partial x}\delta x+\frac{\partial I}{\partial y}\delta y+\frac{\partial I}{\partial t}\delta t\approx 0$$

and by dividing each term by δt, the optical flow constraint equation can be written as follows,

(7) $$\frac{\partial I}{\partial x}\frac{dx}{dt}+\frac{\partial I}{\partial y}\frac{dy}{dt}+\frac{dI}{dt}\approx 0.$$

Resolving the optical flow constraint equation (Equation (7)) consists of the estimation of both parameters $u=\frac{dx}{dt}$ and $v=\frac{dy}{dt}$. Several methods have been proposed to perform this specific task. The authors in [53,54] propose an overview of such approaches. In the current work, dense optical flow is applied, using the method of Farnebäck [50], to compute OFIs from consecutive greyscale images. A depiction of such a sequence of images can be seen in Figure 1c (both motion direction and motion velocity are color encoded).

### 3.3. Network Architecture

**Figure 2:**
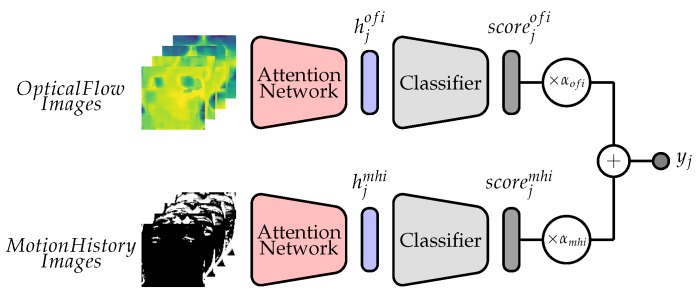
Two-Stream Attention Network with Weighted Score Aggregation.

As opposed to still images, the motion component of a video sequence is integrated into both MHIs and OFIs, therefore providing more valuable information for facial expressions analysis. Therefore, the proposed architecture consists of a multi-view learning [55] neural network with both OFIs and MHIs as input channels. An overall illustration of the proposed two-stream neural network can be seen in Figure 2. In a nutshell, an attention network specific to each input channel (OFIs and MHIs) first generates a weighted representation from the *j*th input sequence ($h_{j}^{ofi}$ and $h_{j}^{mhi}$). The generated representation is subsequently fed into a channel specific classification model (which in this case is a MLP). The resulting class probabilities of each channel ($score_{j}^{ofi}$ and $score_{j}^{mhi}$) are further fed into an aggregation layer with a linear output function, where a weighted aggregation of the provided scores is performed as follows,

(8) $$score_{j}=\alpha_{ofi}\cdot score_{j}^{ofi}+\alpha_{mhi}\cdot score_{j}^{mhi}$$

with the constraint

(9) $$\alpha_{ofi}+\alpha_{mhi}=1.$$

The entire architecture is trained in an end-to-end manner by using the following loss function,

(10) $$L=\lambda_{ofi}\cdot L_{ofi}+\lambda_{mhi}\cdot L_{mhi}+\lambda_{agg}\cdot L_{agg}$$

where the loss functions of each input channel and of the aggregation layer are respectively depicted with $L_{ofi}$, $L_{mhi}$ and $L_{agg}$. The parameters $\lambda_{ofi}$, $\lambda_{mhi}$ and $\lambda_{agg}$ correspond to the regularisation parameters of each respective loss function. Once the network has been trained, unseen samples are classified based on the output of the aggregation layer.

**Figure 3:**
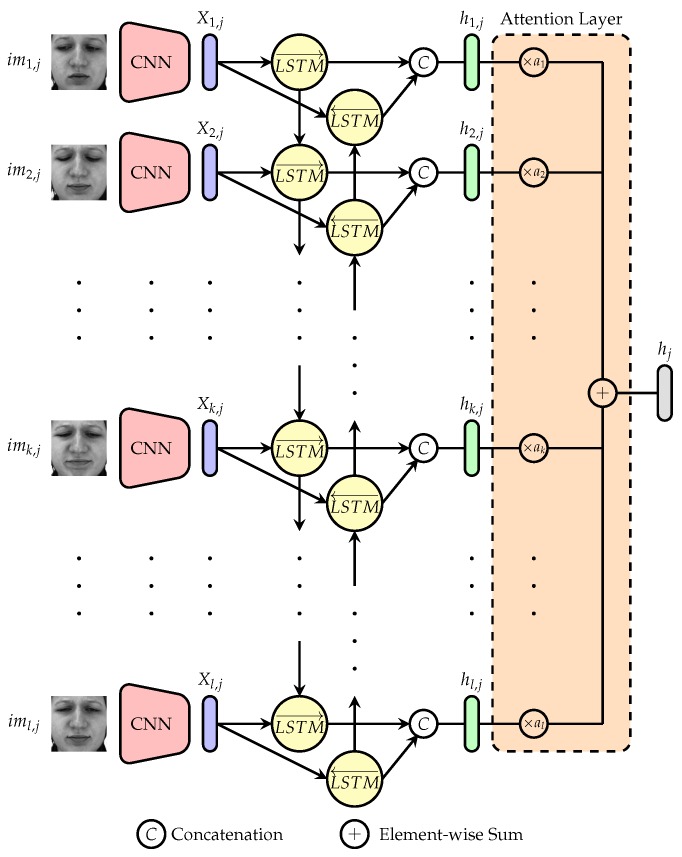
Attention Network.

The attention network (see Figure 3) consists of a CNN coupled to a BiLSTM with a frame attention module [47]. The CNN consists of a time distributed feature embedding network which takes a single facial image $im_{k,j}$ as input and generates a fixed-dimension feature representation $X_{k,j}$ specific to that image. Therefore, the output of the *j*th input sequence of facial images ${\left\{im_{k,j}\right\}}_{k=1}^{l}$ consists of a set of facial features ${\left\{X_{k,j}\right\}}_{k=1}^{l}$. The temporal component of the sequence of images is further integrated by using a BiLSTM layer. A BiLSTM [56] RNN is an extension of a regular LSTM [40] RNN, to enable the use of context representations in both forward and backward directions.

**Figure 4:**
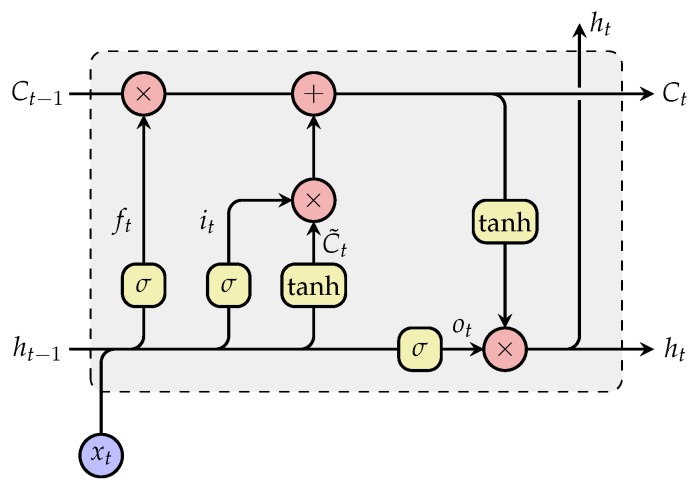
Long Short-Term Memory (LSTM) Recurrent Neural Network (RNN).

It consists of two LSTM layers, one processing the input sequence $\left\{X_{1,j},X_{2,j},\ldots ,X_{l,j}\right\}$ sequentially forward in time (from $X_{1,j}$ to $X_{l,j}$) and the second processing the input sequence sequentially backward in time (from $X_{l,j}$ to $X_{1,j}$). A LSTM RNN is capable of learning long-term dependencies in sequential data, while avoiding the vanishing gradient problem of standard RNNs [57]. This is achieved throughout the use of cell states (see Figure 4), which regulate the amount of information flowing through a LSTM network throughout the use of three principal gates: forget gate ($f_{t}$), input gate ($i_{t}$) and output gate ($o_{t}$). The cell’s output $h_{t}$ (at each time step *t*) is computed, given a specific input $x_{t}$, the previous hidden state $h_{t-1}$, and the previous cell state $C_{t-1}$, as follows,

(11) $$f_{t}=\sigma \left(W_{f}x_{t}+U_{f}h_{t-1}+b_{f}\right)$$

(12) $$i_{t}=\sigma \left(W_{i}x_{t}+U_{i}h_{t-1}+b_{i}\right)$$

(13) $${\tilde{C}}_{t}=tanh\left(W_{c}s_{t}+U_{c}h_{t-1}+b_{c}\right)$$

(14) $$C_{t}=f_{t}⊗C_{t-1}+i_{t}⊗{\tilde{C}}_{t}$$

(15) $$o_{t}=\sigma \left(W_{o}x_{t}+U_{o}h_{t-1}+b_{o}\right)$$

(16) $$h_{t}=o_{t}⊗tanh\left(C_{t}\right)$$

where σ represents the sigmoid activation function $\sigma \left(x\right)={\left(1+exp\left(-x\right)\right)}^{-1}$ and tanh represents the hyperbolic tangent activation function. The element-wise multiplication operator is represented by the symbol ⊗. The weight matrices for the input $x_{t}$ are represented by $W_{i}$, $W_{f}$, $W_{o}$ and $W_{c}$ for the input gate, forget gate, output gate and cell state, respectively. Analogously, the weight matrices for the previous hidden state $h_{t-1}$ for each gate are represented by $U_{i}$, $U_{f}$, $U_{o}$ and $U_{c}$. The amount of information to be further propagated into the network is controlled by the forget gate (Equation (11)), the input gate (Equation (12)) and the computed cell state candidate ${\tilde{C}}_{t}$ (Equation (13)). These parameters are subsequently used to update the cell state $C_{t}$ based on the previous cell state $C_{t-1}$ (Equation (14)). The output of the cell can subsequently be computed using both Equation (15) and Equation (16).

In the current work, the hidden representation stemming from the forward pass $\left\{\vec{h_{1,j}},\vec{h_{2,j}},\ldots ,\vec{h_{l,j}}\right\}$ and the one stemming from the backward pass $\left\{\overset{\leftarrow }{h_{1,j}},\overset{\leftarrow }{h_{2,j}},\ldots ,\overset{\leftarrow }{h_{l,j}}\right\}$ are subsequently concatenated $\left\{\left[\vec{h_{1,j}},\overset{\leftarrow }{h_{1,j}}\right],\left[\vec{h_{2,j}},\overset{\leftarrow }{h_{2,j}}\right],\ldots ,\left[\vec{h_{l,j}},\overset{\leftarrow }{h_{l,j}}\right]\right\}$ and fed into the next layer. For the sake of simplicity, the output of the BiLSTM layer will be depicted as follows, $\left\{h_{1,j},h_{2,j},\ldots ,h_{l,j}\right\}$ (with $h_{k,j}=\left[\vec{h_{k,j}},\overset{\leftarrow }{h_{k,j}}\right]$). The next layer consists of an attention layer, where self-attention weights ${\left\{a_{k}\right\}}_{k=1}^{l}$ are computed and subsequently used to generate a single weighted representation of the input sequence. The self-attention weights are computed as follows,

(17) $$\alpha_{k}=elu\left(W_{k}h_{k,j}+b_{k}\right)$$

(18) $$a_{k}=\frac{exp(\alpha_{k})}{\sum_{i=1}^{l}exp\left(\alpha_{i}\right)}$$

where $W_{k}$ are the weights specific to the input feature representation $h_{k,j}=\left[\vec{h_{k,j}},\overset{\leftarrow }{h_{k,j}}\right]$ and elu represents the exponential linear unit activation function [58], which is defined as

(19) $$elu_{\alpha }\left(x\right)=\left\{\begin{array}{cc} \alpha \cdot \left(\exp(x)-1\right) & \mathrm{if}\;x<0\\ x & \mathrm{if}\;x\geq 0 \end{array}\right.$$

with α=1. Each self-attention weight expresses the relevance of a specific image for the corresponding emotional state expressed within the video sequence. Thereby, relevant images should be assigned significantly higher weight values as irrelevant images. The final representation of the input sequence is subsequently computed by performing a weighted aggregation of the BiLSTM output $\left\{h_{1,j},h_{2,j},\ldots ,h_{l,j}\right\}$ based on the computed self-attention weights ${\left\{a_{k}\right\}}_{k=1}^{l}$ as follows,

(20) $$h_{j}=\sum_{k=1}^{l}a_{k}\cdot h_{k,j}$$

and is further used to perform the classification task.

## 4. Experiments

In the following section, a description of the experiments performed for the evaluation of the proposed approach is provided. First, the datasets used for the evaluation are briefly described, followed by a depiction of the conducted data preprocessing steps. The experimental settings as well as the performed experiments are described subsequently. This section is finally concluded with a description and discussion of the experimental results.

### 4.1. Datasets Description

**Figure 5:**
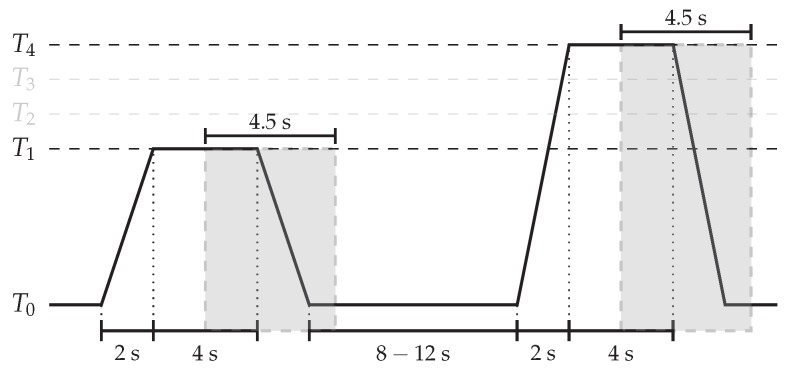
Video Signal Segmentation (BioVid Heat Pain Database (Part A)). Experiments are carried out on windows of length 4.5 s with a temporal shift of 4 s from the elicitations’ onsets.

The presented approach is evaluated on both the *BioVid Heat Pain Database (Part A)* (BVDB) [4] and the *SenseEmotion Database* (SEDB) [6]. Both datasets were recorded with the principal goal of fostering research in the domain of pain recognition. In both cases, several healthy participants were submitted to a series of individually calibrated heat-induced painful stimuli, using the exact same procedure. Whereas the BVDB consists of 87 individuals submitted to four individually calibrated and gradually increasing levels of heat-induced painful stimuli ($T_{1}$, $T_{2}$, $T_{3}$ and $T_{4}$), the SEDB consists of 40 individuals submitted to three individually calibrated and gradually increasing levels of heat-induced stimuli ($T_{1}$, $T_{2}$ and $T_{3}$). Each single level of heat-induced pain stimulation was randomly elicited a total of 20 times for the BVDB and 30 times for the SEDB. Each elicitation lasted 4 s, followed by a recovery phase of a random length of 8 to 12 s during which a baseline temperature $T_{0}$ ($32^{\circ }C$) was applied (see Figure 5). Whereas the elicitations were performed uniquely on one specific hand for the BVDB, the experiments were conducted twice for the SEDB, with the elicitation performed each time on one specific arm (left forearm and right forearm). Therefore, with the inclusion of the baseline temperature $T_{0}$, the dataset specific to the BVDB consists of a total of 87×20×5=8700 samples, whereas the SEDB consists of a total of 40×30×4×2=9600 samples. During the experiments, the demeanour of each participant was recorded using several modalities consisting of video and bio-physiological channels for the BVDB, while the SEDB included audio, video and bio-physiological channels. The current work focuses uniquely on the video modality, and the reader should refer to the work in [10,14,15,16,33,59,60,61,62,63,64] for more experiments including the other recorded modalities.

### 4.2. Data Preprocessing

The evaluation performed in the current work is undertaken in both cases (BVDB and SEDB) on video recordings performed by a frontal camera. The recordings were performed at a frame rate of 25 frames per second (fps) for the BVDB and 30 fps for the SEDB. Furthermore, the evaluation is performed uniquely on windows of length 4.5 s with a shift of 4 s from the elicitation’s onset, as proposed in [16] (see Figure 5). Once these specific windows are extracted, the facial behaviour analysis toolkit OpenFace [65] is used for the automatic detection, alignment and normalisation of the facial area (with a fixed size of 100×100 pixels) in each video frame. Subsequently, MHI sequences and OFI sequences are extracted using the OpenCV library [66]. Both MHIs and OFIs are generated relatively to a reference frame, which in this case is the very first frame of each video sequence. Concerning MHIs, the temporal extent parameter τ (see Equation (1)) was set to the length of the sequence of images (25×4.5≅113 frames for the BVDB and 30×4.5=135 frames for the SEDB). Furthermore, the threshold parameter ξ (see Equation (2)) was set to 1 to capture any single motion from two consecutive frames (in this case, the fluctuation of pixel intensities between the reference frame and the *t*th frame). Finally, to reduce the computational requirements, the number of samples in each sequence is reduced by sequentially selecting each second frame of an entire sequence for the BVDB (resulting in sequences with a total length of 57 frames), and each third frame of an entire sequence for the SEDB (resulting in sequences of length 45 frames). The dimensionality of the tensor input specific to the BVDB is, respectively, $\left(bs,57,100,100,3\right)$ for OFI sequences and $\left(bs,57,100,100,1\right)$ for MHI sequences (bs representing the batch size). The dimensionality of the tensor input specific to the SEDB is, respectively, $\left(bs,45,100,100,3\right)$ for OFI sequences and $\left(bs,45,100,100,1\right)$ for MHI sequences.

### 4.3. Experimental Settings

The evaluation performed in the current work consists of the discrimination between high and low stimuli levels. Therefore, two binary classification tasks are performed for each database: $T_{0}vs.T_{4}$ and $T_{1}vs.T_{4}$ for the BVDB, and $T_{0}vs.T_{3}$ and $T_{1}vs.T_{3}$ for the SEDB. Furthermore, the assessment of the proposed approach is conducted by applying a *Leave-One-Subject-Out* (LOSO) cross-validation evaluation, which means that a total of 87 experiments were conducted for the BVDB (40 experiments for the SEDB), during which the data specific to each participant is used once to evaluate the performance of the classification architecture optimised on the data specific to the remaining 86 participants (the data specific to 39 participants is used to optimise the architecture for the SEDB, and the data specific to the remaining participant is used to evaluate the performance of the architecture).

The feature embedding CNN used for the evaluation is adapted from the one proposed by the Visual Geometry Group of the University of Oxford *VGG16* [67]. The depth of the CNN model is substantially reduced to a total of 10 convolutional layers (instead of 13 as in the *VGG16* model), and the number of convolutional filters is gradually increased from one convolutional block to the next starting from 8 filters until a maximum of 64 filters. The activation function in each convolutional layer consists of the *elu* activation function (instead of the rectified linear unit (*relu*) activation function as in the *VGG16* model). Max-pooling and Batch Normalisation [68] are performed after each convolutional block. A detailed description of the feature embedding CNN architecture can be seen in Table 1. The coupled BiLSTM layer consists of two LSTM RNNs with 64 units each. The resulting sequence of spatio-temporal features is further fed into the attention layer in order to generate a single aggregated representation of the input sequence. The classification is further performed based on this representation and the architecture of the classification model is described in Table 2. The exact same architecture is used for the two input sequences (MHIs and OFIs). The outputs of the classifiers are further aggregated based on both Equation (8) and Equation (9). The whole architecture is subsequently trained in an end-to-end manner, using the Adaptive Moment Estimation (Adam) [69] optimisation algorithm with a fixed learning rate set empirically to $10^{-5}$. The categorical cross entropy loss function is used for each network ($L_{mhi}=L_{ofi}=L_{agg}=L$), and is defined as follows,

(21) $$L=-\sum_{j=1}^{c}y_{j}log\left({\hat{y}}_{j}\right)$$

where ${\hat{y}}_{j}$ represents the classifier’s output, $y_{j}$ is the ground-truth label value and c∈N is the number of classes for a specific classification task.

The regularisation parameters of the loss function in Equation (10) are set as follows: $\lambda_{mhi}=\lambda_{ofi}=0.2$ and $\lambda_{agg}=0.6$. The value of the regularisation parameter specific to the aggregation layer’s loss is set higher than the others in order to enable the architecture to compute robust aggregation weights. The whole architecture is trained for a total of 20 epoches with the batch size set to 40 for the BVDB and 60 for the SEDB. The implementation and evaluation of the whole architecture is done with the Python libraries Keras [70], Tensorflow [71] and Scikit-learn [72].

### 4.4. Results

**Figure 6:**
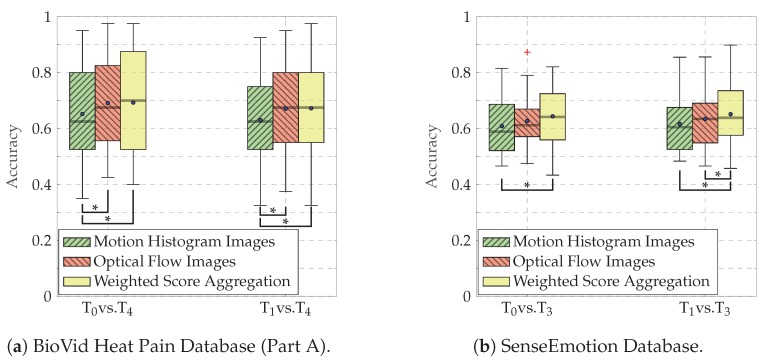
Classification performance (Accuracy). An asterisk (*) indicates a significant performance improvement. The test has been conducted using a Wilcoxon signed rank test with a significance level of 5%. Within each boxplot, the mean and the median classification accuracy are depicted respectively with a dot and a horizontal line.

The performance of the classification architectures specific to each input channel (MHIs and OFIs), as well as the performance of the weighted score aggregation approach are depicted in Figure 6. The performance metric in this case is the accuracy, which is defined as

(22) $$Accuracy=\frac{tp+tn}{tp+fp+tn+fn}$$

where tp refers to true positives, tn refers to true negatives, fp refers to false positives and fn refers to false negatives (since we are dealing with a binary classification task with two balanced datasets). For both datasets and both classification tasks, the aggregation approach significantly outperforms the classification architecture based uniquely on MHIs. Furthermore, the classification architecture based uniquely on OFIs outperforms the one based on MHIs for both databases and both classification tasks, with significant performance improvement in the case of the BVDB. The aggregation approach also performs slightly better than the architecture based uniquely on OFIs for both databases, although not significantly in most cases. The only significant performance improvement is achieved for the classification task $T_{1}$ vs. $T_{4}$ for the SEDB. However, the performance of both channel specific architectures and the performance of the score aggregation approach are significantly higher than chance level (which is 50% in the case of binary classification tasks) pointing at the relevance of the designed approach. Furthermore, the performance of the classification architecture is improved by using both channels and performing a weighted aggregation of the scores of both channel specific deep attention models.

**Figure 7:**
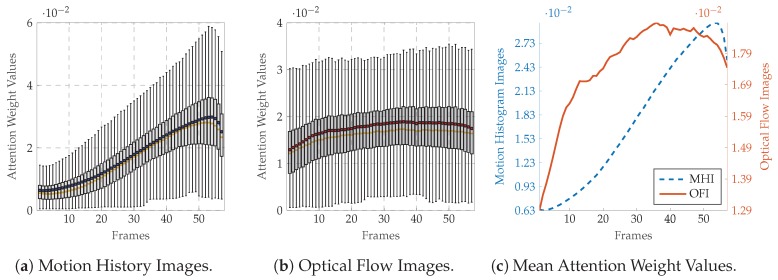
BioVid Heat Pain Database (Part A): Attention network weight values for the classification task $T_{0}vs.T_{4}$. Within each boxplot in (**a**,**b**), the mean and the median weight values are depicted, respectively, with a dot and a horizontal line. In (**c**), the average weight values are normalised between the maximum average value and the minimum average value to allow a better visualisation of the values distributions.

**Figure 8:**
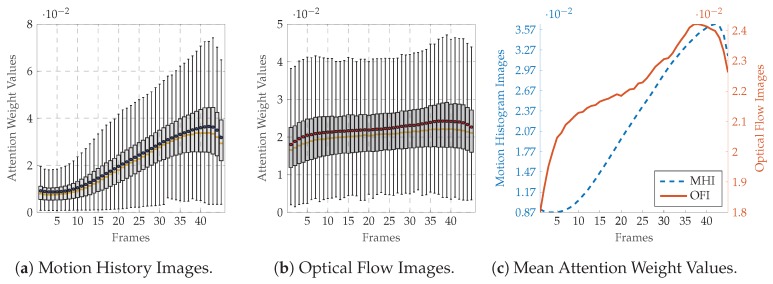
SenseEmotion Database: Attention network weight values for the classification task $T_{0}vs.T_{3}$. Within each boxplot in (**a**,**b**), the mean and the median weight values are depicted respectively with a dot and a horizontal line. In (**c**), the average weight values are normalised between the maximum average value and the minimum average value to allow a better visualisation of the values distributions.

Moreover, to provide more insights into the self attention mechanism, the frame attention weight values computed at each evaluation step during the LOSO cross-validation evaluation process are depicted in Figure 7 for the BVDB and in Figure 8 for the SEDB (uniquely for the classification task $T_{0}$ vs. $T_{4}$, as the results for the classification task $T_{1}$ vs. $T_{4}$ are similar). The distribution of the weight values specific to the MHI deep attention models for both databases (Figure 7a,c for the BVDB, Figure 8a,c for the SEDB) is skewed left. It depicts a steady growth of weight values along the temporal axis of each sequence, with the MHIs located at the end of a sequence weighted significantly higher as the others. This is in accordance with the sequential extraction process of MHIs, as each extracted image contains more motion information as the previous one, with the last images accumulating almost the totality of motion information of an entire sequence. Therefore, concerning the actual classification task, the last MHIs are more interesting and relevant than the early images. Thus, such images should be weighted accordingly higher. The designed network is therefore capable of conducting this specific task by using self attention mechanisms.

A similar observation can be made concerning the distribution of the weight values of OFIs (see Figure 7b,c for the BVDB, Figure 8b,c for the SEDB). Both depicted distributions are also skewed left, with gradually increasing weight values relative to the temporal axis. This shows that the recorded pain-related facial expressions for both BVDB and SEDB consist of gradually evolving facial movements, starting from a neutral facial depiction (not relevant for the actual classification task) to the apex of the facial movement (which is the most relevant frame for the depicted facial emotion) before gradually turning back to the neutral facial depiction. Therefore, the network assigns weight values according to this specific characterisation of pain-related facial movements using attention mechanisms, thus the relevance of such approaches for facial expression analysis.

**Figure 9:**
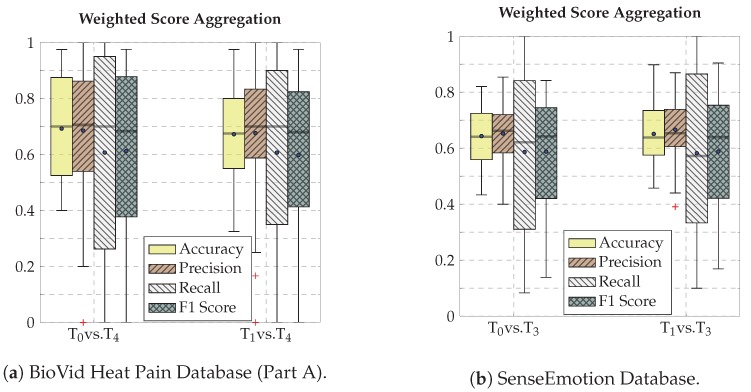
Weighted score aggregation classification performance. Within each box plot, the mean and median values of the respective performance evaluation metrics are depicted with a dot and a horizontal line, respectively.

Furthermore, the performance of the weighted score aggregation approach is further assessed based on the following additional performance metrics,

(23) $$Macro\;Precision=\frac{1}{c}\sum_{i=1}^{c}\frac{tp_{i}}{tp_{i}+fp_{i}}$$

(24) $$Macro\;Recall=\frac{1}{c}\sum_{i=1}^{c}\frac{tp_{i}}{tp_{i}+fn_{i}}$$

(25) $$Macro\;F1\;Score=\frac{2\times Macro\;Precision\times Macro\;Recall}{Macro\;Precision+Macro\;Recall}$$

where $tp_{i}$, $fp_{i}$ and $fn_{i}$ refer, respectively, to the true positives, false positives and false negatives of the *i*th class. The results of the evaluation are depicted in Figure 9, for both the BVDB (see Figure 9a) and the SEDB (see Figure 9b).

These results depict a huge variance amongst all performance metrics, in particular the MacroRecall, which points at the fact that the classification tasks remain difficult. The evaluation on some participants yields a MacroF1Score of null or nearly null, pointing at the fact that the architecture is unable to discriminate between low and high levels of pain elicitation for these specific participants. This is, however, similar and in accordance with previous works on these specific datasets. The authors of the BVDB in [73] were able to identify a set of participants who did not react to the levels of pain elicitation, therefore causing the huge variance in the classification experiments.

Finally, the performance of the weighted score aggregation approach is compared to other pain-related facial expressions classification approaches proposed in the literature. For the sake of fairness, we compare the results of the proposed approach with those results in related works which are based on the exact same dataset and were computed based on the exact same evaluation protocol (LOSO). The results are depicted in Table 3 for the BVDB and in Table 4 for the SEDB.

In both cases, the performance of the weighted score aggregation approach is on par with the best performing approaches. However, it has to be mentioned that the authors of the best performing approaches for both the BVDB [8] and the SEDB [15] perform a subject-specific normalisation of the extracted feature representations in order to compensate for the differences in expressiveness amongst the participants. Although this specific preprocessing step has proven to significantly improve the classification performance of the architecture [61], it is not realistic as it requires that the whole testing set is already available beforehand. The normalisation parameters should be learned on the available training material and subsequently applied to the testing material during the inference phase. Nevertheless, the proposed approach based on the weighted aggregation of the scores of both MHI- and OFI-specific deep attention models generalises well and is capable of achieving state-of-the-art classification performances.

## 5. Conclusions

In the current work, an approach based on a weighted aggregation of the scores of two deep attention networks based, respectively, on MHIs and OFIs has been proposed and evaluated for the analysis of pain-related facial expressions. The assessment performed on both BVDB and SEDB shows that the proposed approach is capable of achieving state-of-the-art classification performances and is on par with the best performing approaches proposed in the literature. Moreover, the visualisation of the weight values stemming from the implemented attention mechanism shows that the network is capable of identifying relevant frames in relation with the current level of pain elicitation depicted by a sequence of images, by assigning significantly higher values to the most relevant images in comparison to the weight values of irrelevant images. Furthermore, as the proposed architecture was trained from scratch in an end-to-end manner, it is believed that transfer learning, in particular, for the feature embedding CNN used to generate the feature representation of each frame, could potentially improve the performance of the whole architecture. Such an analysis was not conducted in the current work, as the optimisation of the presented approach was not the goal of the conducted experiments, but rather the assessment of such an architecture for the analysis of pain-related facial expressions. Moreover, a multi-stage training strategy could also potentially improve the overall performance of the architecture, as the end-to-end trained approach is likely to suffer from overfitting, in particular, when considering the coupled aggregation layer. The representation of the input sequences should be further investigated as well. Both MHIs and OFIs have the temporal aspect of the sequences integrated into their properties. The performed evaluation has shown that a model based on OFIs significantly outperforms the one based on MHIs in most cases. However, it has also been shown that most of the interesting frames in MHI sequences are located at the very end of the temporal axis of each sequence. Therefore, single MHIs extracted from entire sequences could also be used as input for deep architectures. Overall, the performed experiments show that the discrimination between lower and higher pain elicitation levels remains a difficult endeavour. This is due to the variety of expressiveness amongst the participants. However, personalisation and transfer learning strategies could potentially help improve the performance of inference models applied in this specific area of research.

## Figures and Tables

**Table 1:** Feature embedding CNN architecture.

Layer	No. Filters
2× Conv2D	8
MaxPooling2D	−
Batch Normalisation	−
2× Conv2D	16
MaxPooling2D	−
Batch Normalisation	−
3× Conv2D	32
MaxPooling2D	−
Batch Normalisation	−
3× Conv2D	64
MaxPooling2D	−
Batch Normalisation	−
Flatten	−

The size of the kernels is identical for all convolutional layers and is set to 3×3, with the convolutional stride set to 1×1. Max-pooling is performed after each block of convolutional layers over a 2×2 window, with a 2×2 stride.

**Table 2:** Classifier Architecture.

Layer	No. Units
Dropout	−
Fully Connected	64
Dropout	−
Fully Connected	*c*

The dropout rate is empirically set to 0.25. The first fully connected layer uses the *elu* activation function, while the last fully connected layer consists of a *softmax* layer (whereby *c* depicts the number of classes of the classification task).

**Table 3:** Classification performance comparison to early works on the BioVid Heat Pain Database (Part A) in a LOSO cross-validation setting for the classification task $T_{0}vs.T_{4}$.

Approach	Description	Performance
Yang et al. [27]	BSIF	65.17
Kächele et al. [31,62]	Geometric Features	65.55±14.83
Werner et al. [8]	Standardised Facial Action Descriptors	72.40
Our Approach	Motion History Images	65.17±15.49
Our Approach	Optical Flow Images	69.11±14.73
Our Approach	Weighted Score Aggregation	69.25±17.31

The performance metric consists of the average accuracy (in %) over the LOSO cross-validation evaluation. The best performing approach is depicted in bold and the second best approach is underlined.

**Table 4:** Classification performance comparison to early works on the SenseEmotion Database in a LOSO cross-validation setting for the classification task $T_{0}vs.T_{3}$.

Approach	Description	Performance
Kalischek et al. [38]	Transfer Learning	60.10±00.06
Thiam et al. [15]	Standardised Geometric Features	66.22±14.48
Our Approach	Motion Histogram Images	60.86±09.81
Our Approach	Optical Flow Images	62.70±09.24
Our Approach	Weighted Score Aggregation	64.35±10.40

The performance metric consists of the average accuracy (in %) over the LOSO cross-validation evaluation. The best performing approach is depicted in bold and the second best approach is underlined.

## Sample Availability

The BioVid Heat Pain Database (Part A) is publicly available for non-commercial research and can be acquired by contacting the authors of the database at the following web-page: http://www.iikt.ovgu.de/BioVid.print.
